# Analysis of Autofluorescence in Polymorphonuclear Neutrophils: A New Tool for Early Infection Diagnosis

**DOI:** 10.1371/journal.pone.0092564

**Published:** 2014-03-21

**Authors:** Antoine Monsel, Sandrine Lécart, Antoine Roquilly, Alexis Broquet, Cédric Jacqueline, Tristan Mirault, Thibaut Troude, Marie-Pierre Fontaine-Aupart, Karim Asehnoune

**Affiliations:** 1 Multidisciplinary Intensive Care Unit, Department of Anesthesiology and Critical Care, La Pitié-Salpêtrière Hospital, Assistance Publique-Hôpitaux de Paris, Paris, France; 2 UPMC Université Paris, Paris, France; 3 CPBM/CLUPS, université Paris-Sud, Orsay Cedex, France; 4 Laboratoire UPRES EA 3826, Thérapeutiques cliniques et expérimentales des infections, Faculté de Médecine, Faculté de Nantes, Nantes, France; 5 CHU Nantes, Pôle Anesthésie Réanimation, Service d'Anesthésie Réanimation Chirurgicale, Hôtel Dieu, Nantes, France; 6 Vascular Medicine Unit, European Hospital Georges-Pompidou, Assistance Publique-Hôpitaux de Paris, Paris Descartes University, PRES Paris Sorbonne Cité, Paris, France; 7 ISMO, CNRS- Université Paris-Sud, Orsay, France; Charité-University Medicine Berlin, Germany

## Abstract

Diagnosing bacterial infection (BI) remains a challenge for the attending physician. An *ex vivo* infection model based on human fixed polymorphonuclear neutrophils (PMNs) gives an autofluorescence signal that differs significantly between stimulated and unstimulated cells. We took advantage of this property for use in an *in vivo* pneumonia mouse model and in patients hospitalized with bacterial pneumonia. A 2-fold decrease was observed in autofluorescence intensity for cytospined PMNs from broncho-alveolar lavage (BAL) in the pneumonia mouse model and a 2.7-fold decrease was observed in patients with pneumonia when compared with control mice or patients without pneumonia, respectively. This optical method provided an autofluorescence mean intensity cut-off, allowing for easy diagnosis of BI. Originally set up on a confocal microscope, the assay was also effective using a standard epifluorescence microscope. Assessing the autofluorescence of PMNs provides a fast, simple, cheap and reliable method optimizing the efficiency and the time needed for early diagnosis of severe infections. Rationalized therapeutic decisions supported by the results from this method can improve the outcome of patients suspected of having an infection.

## Introduction

Severe bacterial infection (BI) remains a major cause of hospital morbidity and mortality, specifically in intensive care units (ICU). The correlation between mortality and the delay in starting antibiotics has been clearly demonstrated in current literature [1]–[3]. The challenge is therefore to promptly diagnose BI to provide rapid antibiotic treatment in order to improve survival rates.

Another challenge is to reduce the daily overuse of antibiotics in order to reduce both antimicrobial resistance and costs. In this context, the duration of antibiotic treatment has been the subjects of heated debates. The development of rapid techniques for not only diagnosing, but also for monitoring an ongoing infection during antibacterial treatment, is required. These techniques will enable the discontinuation of treatment when appropriate, resulting shorter duration of antibiotic use and a reduction in their overall consumption.

Diagnosis of BI is currently performed by direct Gram staining and bacterial culture. While Gram staining is a rapid procedure [4], its efficacy for diagnosing infection remains poor. Bacterial culture may require at least one day to obtain a bacteriological diagnosis. Recently, new approaches, such as reverse transcriptase-polymerase chain reaction assays [5] or procalcitonin (PCT) [6] dosing have been developed, but these diagnostic tools have not been fully validated to diagnose infections early and accurately and are expensive owing to the sophisticated equipment required. In order to complement these microbiological or biomarker approaches, there is an urgent need to develop rapid, reliable, and cheap methods, easily applied in hospitals and biomedical laboratories, in order to enhance early infection diagnosis and to monitor infection progress faster than what is currently available.

We therefore investigated BI diagnosis by autofluorescence emission of the host's polymorphonuclear neutrophils (PMNs), the first cells recruited from the site of infections. These cells play a crucial role in a pathogen's recognition and destruction. Stimulation of PMNs Gi-coupled G-protein coupled receptors (GPCR) with agonists, such as the N-formyl-L-methionyl-L-leucyl-L-phenylalanine peptide (fMLP) released by the bacterial cell wall at the infection site, leads to activation of the intra-cytoplasmic subunits of the nicotinamine adenine dinucleotide phosphate (NADPH) oxidase [7]. This membrane enzyme catalyzes the oxidation reaction of both NADPH and nicotinamine adenine dinucleotide (NADH) coenzymes by oxygen to produce reactive oxygen species responsible for bactericidal activity [8]. This non-mitochondrial generation of ROS is a massive and fast process known as a respiratory or oxidative burst. The toll-like receptors (TLRs) also play a critical role in the recognition of bacteria. They activate the nuclear transcription factor nuclear factor-kappa B and induce the production of pro-inflammatory cytokines, such as tumor necrosis factor-alpha (TNF-α) [9]–[11]. This massive increase in protein synthesis dramatically modifies the oxydo-reduction metabolism in the cells and, consequently, NADH concentration [12]–[15]. Thus, the host's leukocyte activation could be tracked by measuring the variations in the two reduced pyridine nucleotides, NADH and NADPH, that exhibit a similar visible fluorescence referred to as NAD(P)H.

Several animal studies have used the NAD(P)H autofluorescence signal to distinguish and sort PMNs from other types of cells in either bone marrow [16] or broncho-alveolar lavage (BAL) [17], [18] samples. Elsewhere, *in vitro* studies have been performed on living cells [19]–[21] to demonstrate any impact of triggering human PMNs respiratory burst on their NAD(P)H autofluorescence intensity. To the best of our knowledge, such approach has never be extended to either TLRs signalization pathway activation of PMNs or to *in vivo* BI which could be a promising, non-invasive, rapid and inexpensive tool for early BI diagnosis.

Firstly, an *ex vivo* infection model was developed in human-fixed PMNs specifically stimulated with fMLP and TLR agonists, demonstrating the workability of the NAD(P)H fluorescence signal of the cells. We observed significant changes in the autofluorescence intensity of the stimulated PMNs compared with resting cells. Secondly, the study was carried out using a mouse model, enabling us to assess pneumonia with certainty and to obtain the ascertained negative controls. For this *in vivo* model, changes in autofluorescence intensity signal of fixed PMNs obtained from BAL were observed between the infected and non-infected mice. Finally, the method was extended to BAL obtained from ICU patients, revealing PMNs autofluorescence alterations for patients with ventilator-associated pneumonia (VAP).

## Materials and Methods

### Ethics statement

This work is part of a global study on ICU-induced inflammatory dysfunction. In this setting, all the studies involving a biological sampling at our institution were recorded and were declared to the French Ministry of Health in 2008 (authorization number: AC 2008–433) after approval by the institutional review board of Angers (19-12-08). ICU patients were ventilated and sedated when samples were collected. For the global study written informed consent was mandated from a next-of-kin. Considering the present study, BAL samples for PMNs analysis were collected from residual BAL after completion of routine follow-up. The institutional review board of Nantes (N°DC-2011-1399) waived written inform consent and authorized verbal informed consent from relatives and retrospective information was provided to patients. Healthy donors provided written informed consent before blood sampling. The total blood volume collected from each volunteer was 25 mL.

Concerning the animal *in vivo* model, the committee of animal ethics of the University of Nantes approved all of the animal experiments in this study.

### Human cell culture and stimulation

PMNs were isolated from whole blood of healthy donors by centrifugation over a density gradient of Lympholyte-Poly (Cedarlane Tebu-bio, Le Perrey-en-Yvelines, France). Two distinct cell rings were obtained, and the PMNs were taken from the lower ring. PMNs were then centrifuged and resuspended in 0.45% NaCl, washed twice, and incubated at 37°C in 4-well Lab-TekTM slides (Nunc, Roskilde, Denmark) at a concentration of 8×105 cell/ml in RPMI (Invitrogen, Paisley, United Kingdom) supplemented with 4% human AB serum, penicillin G (100 U/ml) and streptomycin (100 μg/ml) (Valbiotech, Paris, France). Lab-Tek™ slides were preferentially used regarding the need to regularly monitor PMN cell cultures with an inverted microscope as well as the optimal adherence, viability and seeding quality of the neutrophils previously noted on glass coverslip chambers.

After 24 hours, to mimic BI, PMNs were stimulated 90 minutes with 50 μg/mL of purified lipopolysaccharide (LPS) from *Escherichia coli* (Sigma, St. Louis, MO) to activate the TLR4 pathway; with 10 μg/mL of the synthesized peptidoglycan Pam3Csk4 (InvivoGen, San Diego, USA) to activate the TLR2 pathway; or with 10^−6^ mol/L of the peptide fMLP (Sigma-Aldrich, St Quentin Fallavier, France) to activate a TLR independent signaling pathway GPCR. For the control samples, PMNs were not stimulated.

### Fixed human cellular sample

To make our *ex vivo* model available as a tool for routine diagnosis, we optimized the study for fixed cells. Thus at the end of all activation conditions (t = 90 minutes, which corresponds to the activation time of cytokine production), supernatants were discarded and 5×10^5^ PMNs were gently resuspended by smooth pipetting, cytospined on glass slides, fixed for 10 minutes with 4% paraformaldehyde (PFA), and mounted with ProLong Gold antifade reagent (Invitrogen, Paisley, United Kingdom). Under these conditions, PMNs remained stable and readable for autofluorescence over several months for slides kept in the dark at 4°C.

### Human cytokine assay

Neutrophils from healthy donors were cultured for 90 minutes in 24-well plates (1×10^6^ cells/well) in medium (RPMI + fetal bovine serum 10%) alone (control, C), or with LPS (*E. coli* O111:B4, 1 μg/ml; Sigma), Pam3Csk4 (1 μg/ml; Sigma), or fMLP (1 nM; Sigma) in a 5% CO2 incubator at 37 °C. Supernatants were collected by centrifugation at 12000 × g for 2 minutes and stored at −80°C before cytokine determination by enzyme linked immuno-sorbent assay kits according to manufacturer instructions (R&D Systems, Abingdon, United Kingdom).

### Alveolar lavage from pneumonia and LPS-induced acute lung injury (ALI) model in mice

Male BALB/cJ (20–24 g) mice were purchased from Janvier Laboratories, Laval, France. Mice were maintained on a 12-hours light/dark cycle with free access to food and water. Methicilline-susceptible *Staphylococcus Aureus* strains (ATCC 29213) and *Pseudomonas aeruginosa* (ATCC 51675) were grown for 18 hours in tryptic soy broth at 37 °C. Immediately before use, cultures were washed twice, with centrifugation at 1000×g for 10 minutes at 37 °C, and then diluted in sterile isotonic saline to be calibrated by nephelometry (1×10^10^ and 1×10^9^ Colony Forming Unit (CFU)/ml for *Staphylococcus aureus* and *Pseudomonas aeruginosa*, respectively). Bacterial concentration (CFU/ml) was controlled by quantitative culture. Pneumonia and LPS-induced ALI model were induced according to previously published methods [22], [23]. Briefly, mice were anesthetized with isoflurane and placed in dorsal recumbency. Transtracheal insertion of a 24-gauge feeding needle was used to inject 70 μl either the bacterial preparation or LPS (*E.Coli* O111:B4, 10 mg/Kg; Sigma). The rate of intratracheal inoculation reaches 100% with this procedure. The mice were euthanized 24 hours after tracheal instillation. Lungs were flushed with 1 ml of saline to obtain BAL fluids and 500 μl were cytospined on glass slides (6 minutes at medium speed), fixed 10 minutes with 4% PFA, and mounted with ProLong Gold antifade reagent (Invitrogen, Paisley, United Kingdom) for further autofluorescence intensity signal analysis.

Total cell numbers in each BAL were counted using a Coulter counter (Beckmann Coulter, Fullerton, CA, USA) and differential cell counts were performed on cytospin preparations stained with Giemsa. The percentage of PMNs in BALs, defined as Gr-1^pos^CD11b^pos^ cells, was determined by flow cytometry analysis (BD Bioscience, LSR-II). Monoclonal antibodies used for flow cytometry were obtained from BD Biosciences (Le Pont de Claix, France): anti-CD11b (M1/70), and from eBiosciences (San Diego, CA, USA): anti-Gr-1 (RB6-8C5). The percentage of PMNs in BAL was also performed by using Ly6G [24]. Data were analyzed using FlowJo software (Treestar, USA).

### Alveolar lavage from patients with ventilated-associated pneumonia (VAP)

Pneumonia was considered when at least 2 signs (body temperature >38°C; purulent pulmonary secretions; leukocytosis >12000/mL or leukopenia <4000/mL) were associated with the appearance of a new infiltrate or changes in an existing infiltrate on chest X-ray. When VAP was suspected, the ICU physician performed a BAL in the affected region of the lung, identified from a chest radiograph, to confirm the diagnosis. The presence of squamous epithelial cells, bronchial cells and bacteria were noted following cytocentrifugation (1500 rpm, 15 mn) and a Gram stain. Fifty μL of BAL (without dilution) were plated on Columbia 5% sheep-blood agar, and on chocolate PolyViteX agar, using a spiral system. All plates were incubated at 37°C under 5% CO2, for 24 h or 48 h. A quantitative BAL was defined as positive if there were more than 10^4^ CFU/mL [25], [26]. The final diagnosis of VAP was based on a Clinical Pulmonary Infection Score ≥6 [27]. BAL fluids (500 μl) were cytospined on glass slides (6 minutes at medium speed), fixed for 10 minutes with 4% PFA, and mounted with ProLong Gold antifade reagent Invitrogen, Paisley, United Kingdom) for further autofluorescence intensity signal analysis.

### Fluorescence imaging

Fluorescence images were first performed using a TCS SP5 AOBS (Leica Microsystems; implemented at the Centre de Photonique Biomédicale of Orsay). Cells were observed using a 63x, 1.4 numerical aperture plan Apochromat oil immersion objective. An Argon ultraviolet laser with a maximum wavelength of 364 nm (1.3 mW mesured at the back focal plan of the objective) was used as the excitation source for the cell endogenous NAD(P)H. Fluorescence was collected in the 400–550 nm range. The flavin fluorescence, which was tested with an Argon laser with maximum wavelength of 488 nm, appeared to be insignificant in these immune cells (data not shown). The resolution of the confocal images was 512×512 pixels (corresponding to an area of ∼250×250 μm^2^), recorded on 12 bits with a zoom value of one. Each image corresponds to an average of four scans. The quantification of the fluorescence intensity was carried out with LASAF software (Leica Microsystems, Wetzlar, Germany) using Region of Interest (ROI) over isolated cells present in the field of each image (approximately 10 ROI per image). Each ROI was carefully outlined by avoiding any area presenting multi-layer superoposed cells. Data were collected in three to five independent experiments for each condition. For the mouse and human BAL study, fluorescence intensity analyses were carried out by two different trained operators blinded to mouse or patient status.

Further fluorescence images were obtained using a conventional right epifluorescence microscope (Olympus BX 51, Tokyo, Japan) equipped with a continuous mercury-arc lamp (X-cite 120Q, EXFO) for excitation and a monochrome cooled CCD camera (CCD XM10 camera, Olympus) for fluorescence emission detection through a 40x objective. The resolution of the images was 1376×1038 pixels (corresponding to an area of ∼220×170 μm^2^), recorded on 14 bits. Fluorescence images were analyzed similarly to confocal images but using ICY software (Institut Pasteur, Paris, France).

### Statistical analysis

Results are expressed as mean ± Standard Deviation (SD) if the data were normally distributed and median interquartile range if not. Normality of the distribution was verified by the Shapiro-Wilk test and scattered dot plots.An ANOVA with a Student *t*-test using Bonferroni's correction for post-hoc analysis was performed for multiple comparisons if the data were normally distributed. If not, a Kruskal Wallis test with Mann-Whitney test using Bonferroni's corrections for post-hoc analysis was used. A receiver-operating curve was built by plotting the sensitivity (true positive rate) as a function of the false positive rate (100-specificity) at different fluorescence intensities. The threshold associated with the best relationship between sensitivity and specificity was determined according to the Youden Index. A value of p<0.05 was considered statistically significant. All statistical analysis was performed using SPSS 13.0 software (Chicago, IL, USA).

## Results

### Autofluorescence intensity from human *ex vivo* stimulated PMNs is different from resting PMNs

PMNs, which are critically involved in the host defense against pathogens, were stimulated with the TLR4 and TLR2 agonists (LPS and Pam3Csk4, respectively) and a GPCR agonist (fMLP).

**Figure 1:**
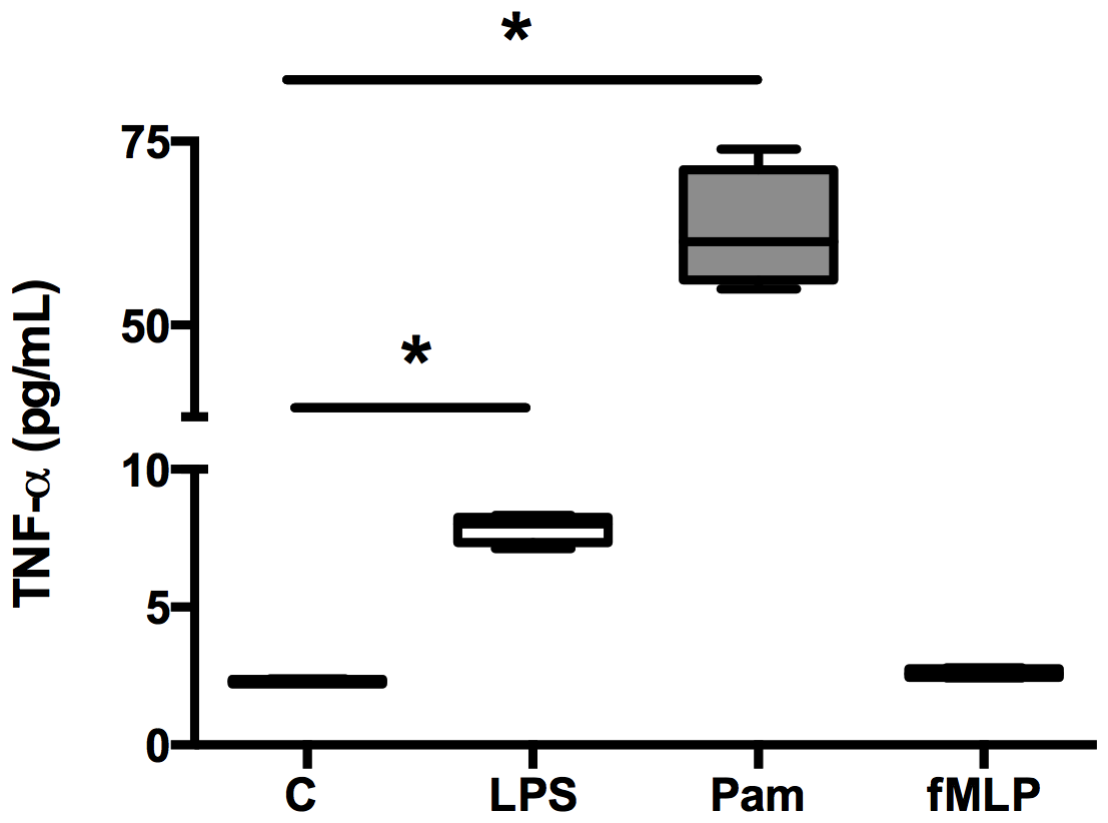
*Ex vivo* production of tumor necrosis factor alpha (TNF-α) by human neutrophils. The release of TNF-α from non-stimulated (control, C) neutrophils, or LPS- (LPS), Pam3Csk4- (Pam), and fMLP- (fMLP) stimulated neutrophils after 90 minutes were measured with enzyme-linked immunosorbent assay. Boxes represent median (interquartile range). Kruskal-Wallis test with *post-hoc* analysis by Mann-Whitney test using Bonferroni's corrections were used to compare responses with and without PRRs ligands. *p<0.05. These data are representative of two different experiments.

We first verified that these agonists effectively stimulated PMNs by measuring TNF-α production after stimulation and compared it with resting PMNs (Figure 1). As expected, TLR agonists (LPS and Pam3Csk4), but not fMLP, induced a TNF-α production in PMNs when compared with untreated PMNs.

**Figure 2:**
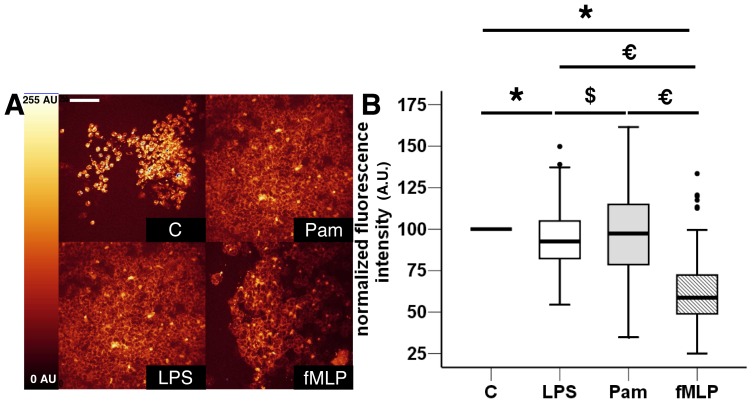
Representative fluorescence intensity variations between *ex vivo* stimulated and non-stimulated cytospined PMNs. A) Representative images of autofluorescence intensities of non-stimulated PMNs (control C), and LPS-, Pam3Csk4- (Pam), and fMLP-stimulated cells (Scale bar, 50 μm). B) The results are represented as boxplots of normalized fluorescence intensity to graphically depict the groups of numerical data for each stimulation condition (n = 1205 studied PMNs from eight healthy volunteers). Boxes represent median (interquartile range). ANOVA with post-hoc analysis by *t*-test using Bonferroni's correction was used to compare autofluorescence levels between each group. *: p<0.001 stimulation groups versus control group, *: p<0.001 fMLP group versus LPS and Pam, €: p<10^−4^ fMLP group versus Pam3Csk4 group, €: p<10^−4^ fMLP group versus Pam3Csk4 and LPS, $: p = 0.004 Pam3Csk4 group versus LPS group.

Eight healthy volunteers were sampled to study 1205 fixed PMNs. Typical overall fluorescence intensity images of the three stimulated and control PMNs groups are illustrated Figure 2 A. The autofluorescence mean values of LPS (93.6±16.2 AU) and fMLP (61.1±18.6 AU) stimulated PMNs were significantly lower than the normalized baseline autofluorescence value of unstimulated PMNs (100 AU; Figure 2 B). For the Pam3Csk4-stimulated PMNs group, the fluorescence variation was not significant. We noted that the decrease of the mean fluorescence intensity was much greater for cells stimulated by fMLP (∼40% fluorescence decrease compared with the control group).

### Autofluorescence intensity in cytospined BAL from pneumonia mouse model samples is different from control samples

**Figure 3:**
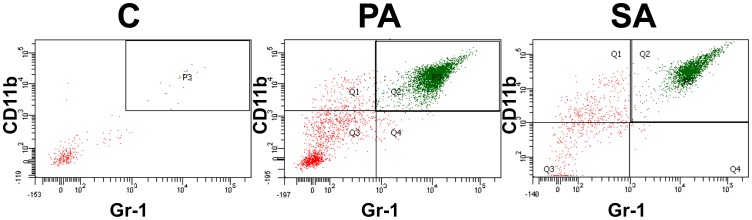
Neutrophils relative proportion determination by flow cytometry study. Example of representative gating obtained by flow cytometry of BAL collected from non-infected mice (PBS treatment: control, C) or pneumonia mouse model 24 hours after tracheal instillation of *Pseudomonas aeruginosa*, PA; or *Staphylococcus aureus*, SA. For this experiment, neutrophils were considered as Gr-1_pos_ CD11b_pos_ cells.

**Figure 4:**
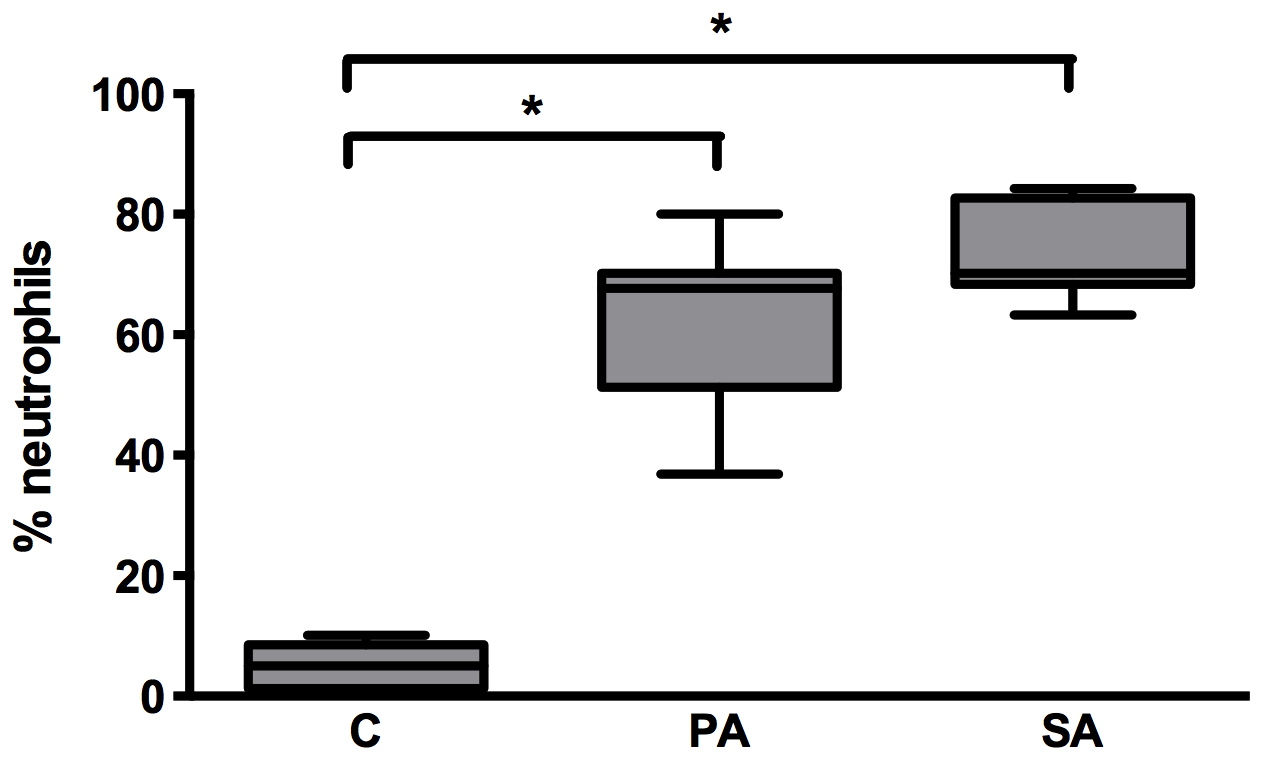
Evaluation of the neutrophil population in control and *Pseudomonas aeruginosa-*or *Staphylococcus aureus*-induced pneumonia mice. Proportions of neutrophils (Gr-1^pos^ CD11b^pos^) were assessed in murine BALs 24 hours after intra-tracheal instillation of saline solution (C), *Pseudomonas aeruginosa* (PA) or *Staphylococcus aureus* (SA). Boxes represent median (interquartile ranges) from three independent experiments (n≥5 mice per group). A Kruskal-Wallis test with Mann-Whitney test using Bonferroni's corrections for post-hoc analysis was used. *: p<0.001 compared with the sham group.

Validation of the pneumonia mice model was previously described by Roquilly et al. [22], [28]. Flow cytometry analyses were performed on cells obtained from BAL (*Pseudomonas aeruginosa* and *Staphylococcus aureus* pneumonia models; Figure 3). We found the presence of 67.7% (51.3–70.2%) and 70.2% (68.8%–84.3%) of neutrophils respectively compared with 5.0% (1.3%–8.6%) of neutrophils in the control group (Figure 4). Using Ly6G, the best marker to discriminate neutrophils in a population of myeloid cells [24], we confirmed that neutrophils were the major cell population in BAL from infected mice with *Pseudomonas aeruginosa* and *Staphylococcus aureus*: 64.2% (53.7–76.9%) and 81.3% (68.1%–84.9%) of neutrophils respectively compared with 7.0% (1.4%–10.1%) of neutrophils in the control group (Figure S1). These data indicated that the majority of the leukocytes studied in BAL cytospined samples were neutrophils, which correlated with a classical bacterial pneumonia observed in the patients.

**Figure 5:**
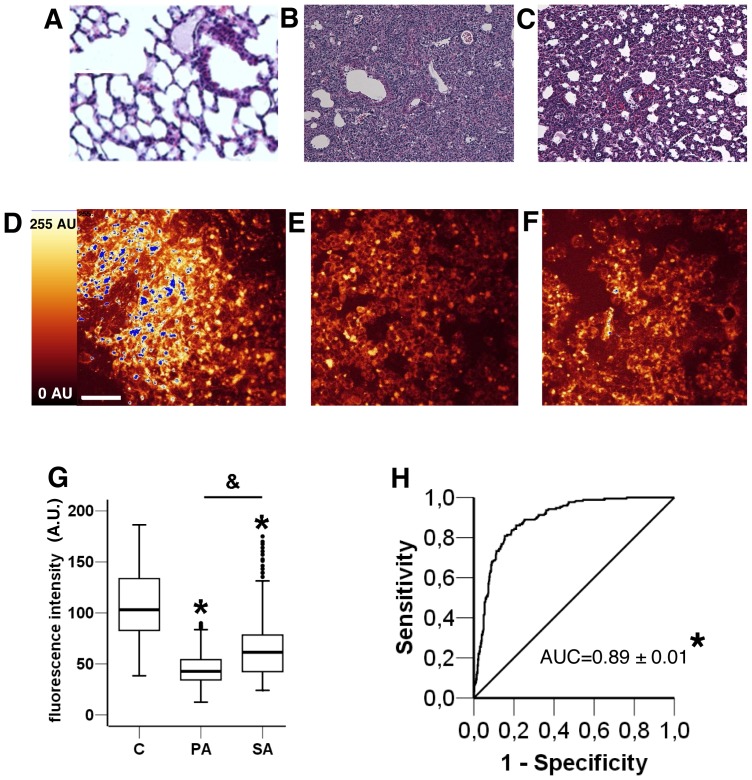
Fluorescence intensity and histology images of BAL in control and *Pseudomonas aeruginosa*- or *Staphylococcus aureus*–induced pneumonia mice. Control condition histology (A) and fluorescence intensity imaging (D); *Staphylococcus aureus*-induced pneumonia histology (B) and fluorescence intensity imaging (E); *Pseudomonas aeruginosa*-induced pneumonia histology (C) and fluorescence intensity imaging (F). Scale bar, 50 μm. (G) Boxplot representation of fluorescence intensity in arbitrary units (I (AU)) of BAL for non infected mice (control, C) and *Pseudomonas aeruginosa* (PA) or *Staphylococcus aureus* (SA) induced pneumonia in mice. Boxes represent median (interquartile range). Data are representative of two independent experiments (Control group, n = 8; *Pseudomonas aeruginosa* group, n = 9; *Staphylococcus aureus* group, n = 12). ANOVA with post-hoc analysis by *t*-test using Bonferroni's correction was used to compare autofluorescence levels between each group. *: p<10^−4^ *Pseudomonas aeruginosa* and *Staphylococcus aureus* groups versus control group. &: p<10^−4^ *Pseudomonas aeruginosa* group versus *Staphylococcus aureus* group. BAL and background noise intensities are different in each condition (p<10^−4^). (H) Receiver operating curve of fluorescence intensity of cells between controls and induced-infectious pneumonia in mice. AUC ± Standard Error: area under curve. *p<10^−4^.

Typical histological lung damage and autofluorescence images of BAL cytospined cells from *Pseudomonas aeruginosa* or *Staphylococcus aureus* pneumonia mouse models are shown in Figure 5. Histological analysis of lung tissue showed thin-walled air spaces with a single pneumocyte layer observed in control mice (Figure 5 A) compared with an alveolar layer thickening and immune cell infiltration after *Staphylococcus aureus* (Figure 5 B) or *Pseudomonas aeruginosa* (Figure 5 C) infection. Twenty-nine mice were sampled to study the autofluorescence of 599 cells. The mean autofluorescence value for the cytospined cells from BAL after *Pseudomonas aeruginosa* pneumonia (Figure 5 E) was significantly lower than the value after *Staphylococcus aureus* (Figure 5 F) pneumonia (44.8±16.3 AU and 66.3±30.6 AU respectively). Both were significantly lower (from 40 to 60%) than the mean autofluorescence value of the cytospined cells from BAL in control mice (107.5±33.9 AU; Figure 5 G).

The receiver operating curve for fluorescence intensity for cells from non-infected and infected BAL mice showed an area under curve of 0.89±0.01 (p<10^−4^; Figure 5 H). As best cut-off for the diagnosis of pneumonia, the Youden index identified an autofluorescence intensity of 75.5 AU (Sensitivity 84%, Specificity 81%).

### The autofluorescence intensity in cytospined BAL from pneumonia mouse model samples is different from LPS-induced ALI samples

**Figure 6:**
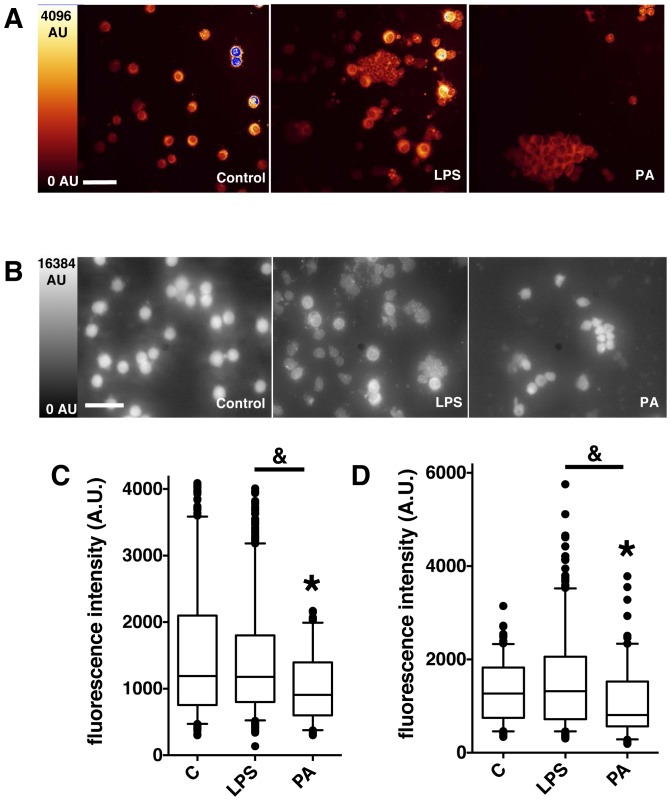
Representative fluorescence intensity images and values in control, LPS-induced acute lung injury and *Pseudomonas aeruginosa*-induced pneumonia in mice. Fluorescence intensity imaging by confocal microscopy (A) and standard epifluorescence microscopy (B) of cytospined BAL samples from Control, LPS-induced acute lung injury (LPS) and *Pseudomonas aeruginosa*-induced pneumonia (PA) in mice. Scale bar, 50 μm. Boxplot representation of fluorescence intensity in arbitrary units (I (AU)) of BAL samples in the 3 different conditions (Control (C), LPS, PA), from confocal images (C) and classical epifluorescence images (D). Boxes represent median (interquartile range). Control group, n = 6, LPS group, n = 7, *Pseudomonas aeruginosa* group, n = 2. Group comparisons were analyzed by Kruskall-Wallis test with a Mann-Whitney test using Bonferroni's corrections for post-hoc analysis. (C) *: p<10^−4^ *Pseudomonas aeruginosa* group versus control group. &: p<10^−4^ *Pseudomonas aeruginosa* group versus LPS group. (D) *: p<0.001 *Pseudomonas aeruginosa* group versus control group. &: p<10^−4^ *Pseudomonas aeruginosa* group versus LPS group.

The murine intratracheal LPS-induced ALI model has been previously validated [23]. This model generates a maximal PMNs accumulation in airways and histological injury 24 hours after LPS instillation. In this separate set of experiments (Figure 6), 15 mice were sampled to study the autofluorescence of 1580 cells. The mean autofluorescence intensity of the cytospined cells from BAL samples was significantly lower in the *Pseudomonas aeruginosa* group than either the control or the LPS-induced ALI groups (909 [600–1398] AU *versus* 1190 [755.5–2098] AU and 1180 [801–1800] AU, respectively; Figure 6 C). No difference was observed between the control and the LPS-induced ALI groups (Figure 6 C).

These assays were first performed with a confocal microscope and duplicated using a standard epifluorescence microscope. Even with this classical-resolution microscope a very good fluorescence contrast was observed between the different cells (figure 6 B) and statistical analysis confirmed that the mean fluorescence value of the cytospined cells from BAL with *Pseudomonas aeruginosa* pneumonia was ∼20–30% lower than for BAL from control or LPS-induced ALI mice (Figure 6 D).

### Autofluorescence intensity in cytospined BAL from human VAP

**Figure 7:**
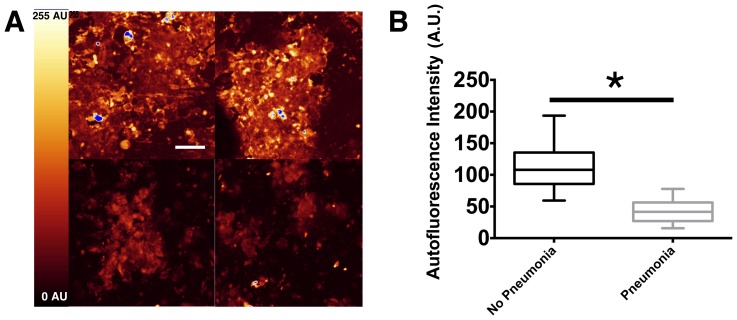
Representative fluorescence intensity images and values in patients with and without VAP. Nine patients were successively screened. Pneumonia was considered when at least two signs (body temperature >38°C, purulent pulmonary secretions, and leukocytosis >12000/mL or leukopenia <4000/mL) were associated with the appearance of a new infiltrate or changes in an existing infiltrate on chest x-ray. BAL was performed to confirm the diagnosis. The final diagnosis of VAP was based on a clinical pulmonary infection score ≥ 6. A) Representative images of autofluorescence intensities of cytospined BAL from patients without VAP (upper panels) and with VAP (lower panels). Scale bar, 50 μm. B) The results are represented as boxplots of fluorescence intensity to graphically depict the groups of numerical data for each group of patients (n = 169 studied ROI from 2 patients without VAP, n = 551 studied ROI from 7 patients with VAP). Boxes represent median (interquartile range). Student's *t*-test was used to compare autofluorescence intensity between both groups. *: p<0.0001 VAP patients (pneumonia) versus non-VAP patients (no pneumonia).

We analyzed the BAL from nine ventilated ICU patients in order to validate our results in a clinical setting. The general characteristics of patients are presented in Table 1. The median clinical pulmonary infection score (Table 2) was 7 (6–9) on the day of BAL sampling. Typical autofluorescence images of cytospined cells from patients without VAP and with VAP are shown in Figure 7 A. Next, 720 ROI were quantified, revealing a mean autofluorescence intensity value of the cytospined cells from VAP patients that was 2.7-fold lower than from patients without VAP (42.4±20.3 AU and 115.2±39.5 AU, respectively; Fig 7 B). The region ROI quantification was blinded to the VAP diagnosis.

**Table 1:** Characteristics of ICU patients (with and without VAP) in which BAL was performed to evaluate neutrophil autofluorescence.

	N = 9
Age, years *– median (interquartile range)*	42 (37–64)
Male – *N (%)*	6 (67)
Simplified acute physiology score II – *median (interquartile range)*	50 (40–57)
Initial condition – *N (%)*	
Trauma	6 (67)
Sepsis	2 (22)
Respiratory failure	1 (11)
Clinical Pulmonary Infection Score – *median (interquartile range)*	7 (6–9)
Pathogens – *N (% of VAP)*	
* Staphylococcus aureus*	2 (22)
* Streptococcus pneumoniae*	3 (33)
* Haemophilus influenzae*	1 (11)
* Enterobacter spp*	2 (22)
* Oropharyngeal flora*	1 (11)
* No pathogens in culture*	1 (11)
Duration of mechanical ventilation, days – *median (interquartile range)*	13 (7–20)
ICU length of stay, days – *median (interquartile range)*	17 (8–37)
Death in ICU, *N (%)*	2 (22)

**Table 2:** Clinical pulmonary infection score.

**Temperature (°C)**	
≥ to 36.5°C and, or equal to 38.4°C	0 point
≥ to 38.5°C and, or equal to 38.9°C	1 point
≥ to 39°C and, or equal to 36°C	2 points
**Blood leukocytes, (/mm^3^)**	
≥4,000 and, ≤ to 11,000	0 point
<4,000 or >11,000	1 point
and if band forms ≥ to 50%	1 point
**Tracheal secretions**	
Absence of tracheal secretions	0 point
Presence of nonpurulent tracheal secretions	1 point
Presence of purulent tracheal secretions	2 points
**Oxygenation**	
Ratio PaO2/FIO2 >240 or acute respiratory distress syndrome	0 point
Ratio PaO2/FIO2 ≤ equal to 240 and no acute respiratory distress syndrome	2 points
**Pulmonary radiography**	
No infiltrate	0 point
Diffuse (or patchy) infiltrate	1 point
Localized infiltrate	2 points
**Progression of pulmonary infiltrate**	
No radiographic progression	0 point
Radiographic progression	2 points
**Culture of tracheal aspirate**	
Pathogenic bacteria cultured in rare or light quantity or no growth	0 points
Pathogenic bacteria cultured in moderate or heavy quantity	1 point
Same pathogenic bacteria seen on Gram stain, add 1 point	1 point

## Discussion

After a BI, PMNs are the first and largest population of immune cells present in the blood and also in the lungs in the case of pneumonia [29]. In the current study, we took advantage of NAD(P)H autofluorescence modifications observed in PMNs to validate a new tool for diagnosing BIs. Furthermore, we demonstrated that this PMNs autofluorescence signal alteration is specific to pneumonia, making it possible to distinguish bacteria-induced inflammation from a non-infectious lung injury.

Our *ex vivo* model using stimulated fixed and mounted human PMNs showed that the autofluorescence signal displayed significant decreases when PMNs were challenged with fMLP or TLR agonists compared with resting cells, revealing cell metabolism modifications after stimulation. PMNs are known to have a very high level of oxidative burst potency, mainly relayed by the NAD(P)H oxidase function [7], [8]. Thus, the main effect of the stimulation of PMNs by pathogens, via fMLP instead of TLRs agonists, can be reasonably explained by significant depletion in cytosolic NAD(P)H concentration which correlates with the observed decrease in autofluorescence intensity. In agreement with our results, it has already been demonstrated that GPCR engagement results in little or no cytokine release in neutrophils. Interestingly, previous *in vitro* studies carried out on human PMNs exposed to either phorbol diester [19] or fMLP [21], as respiratory burst triggers, reported similar results. Using either a flow cytometry [19] or epi-fluorescence microscopy method [21], both studies demonstrated a drop in the NAD(P)H autofluorescence intensity of such stimulated-PMNs. However, another study found a 2-fold increase in NAD(P)H autofluorescence intensity of human PMNs activated *in vitro* with fMLP, compared with resting neutrophils [20]. Several remarkable differences and limitations in the experimental settings could account for these discrepancies. In particular, PMNs stimulation could lead to cell clumping [30], and experiments by flow cytometry or spectrofluorometry using high cell concentrations could therefore result in a disruption in fluorescence signal processing. Autofluorescence intensity quantification based on cell imaging allowed us to precisely acquire the signal from individual cells, ruling out cell superposition. In an attempt to validate the results of our *ex vivo* model, we assessed the cellular autofluorescence alterations in a validated mouse model of pneumonia [22], [28]. This model closely resembles what happens in the clinical scenario, and permitted us to eliminate any false positive or negative values. In this context, *Staphylococcus aureus* (Gram-positive bacteria) and *Pseudomonas aeruginosa* bacteria (Gram-negative bacteria) were chosen because they are critically involved in many human community and nosocomial infections. We first clarified the cell population nature in BAL samples. Although some consensus exists on the very low or absence of autofluorescence properties of lymphocytes [16], [17], conflicting data remain about either the relative monocyte [16]–[18], [20] or eosinophil [17], [31] autofluorescence intensity compared with the neutrophil autofluorescence signal. Considering the present results, we used LY6G in animal experiments, the most specific antibody to isolate neutrophils among myloid cells [24]. Also, in human BAL, neutrophils are by far the largest population of cells, and their morphology is characteristic. The mean autofluorescence intensity variation caused mainly by PMNs from infected animals, as revealed by flow cytometry analysis, was significant enough to distinguish a cytospined BAL from a control mouse from a cytospined BAL from a mouse with pneumonia. These data confirmed the results obtained using the *ex vivo* model, which showed a decrease in fluorescence in the host cells challenged by fMLP or LPS compared with resting cells.

We further investigated wether or not the PMNs autofluorescence level could distinguish an infection-triggered from a non-septic lung inflammation. The LPS-induced ALI model was chosen because this procedure is reproducible, well validated and strongly featured in the literature [23]. LPS is specific to Gram-negative bacteria, and was therefore an accurate positive control for our *Pseudomonas aeruginosa* pneumonia model as infectious model. The results suggest that our technique remains capable of discriminating the autofluorescence intensity level of pneumonia from a non-infectious ALI. Furthermore, no difference was found in PMNs autofluorescence signal of BAL samples between the ALI and control groups, which means that in the context of pneumonia diagnosis, non-infectious ALI could not be detected as a false positive.

After validation of these models, we tested our new tool for diagnosing infections in a clinical setting. For this purpose, PMNs from BAL harvested in mechanically ventilated patients suspected of having VAP were used. VAP is a leading cause of death in ICU patients. Its diagnosis is particularly challenging for the attending physician since current criteria are neither sensitive nor specific [32]. Thus, there is an urgent need to provide new diagnostic tools to complement common clinical and bacteriological indicators. Our results demonstrate that a major difference in the mean autofluorescence intensity of human cells leads to the differentiation of cytospined BALs collected from patients with and without VAP. Thus, as far as VAP is concerned, we could imagine that the mean autofluorescence intensity of a cytospined BAL could help to confirm or to rule out the final diagnosis of VAP in ICU patients. To our knowledge, no studies have previously reported a potential use of PMNs NAD(P)H autofluorescence signal in human sepsis setting. Interestingly, in a flow cytometry study in blood PMNs, Kim *et al.* reported that flavin adenine dinucleotide fluorescence level chemically enhanced by Zn^2+^ dipicolylamine, could distinguish cells with from those without toxic granulations with a sensitivity of >90% and a specificity of >80% [33]. However, since toxic granules can be found in multiple neutrophil abnormalities and diseases distinct from infection, detecting them for improving sepsis diagnosis remains questionable.

A tool for the early diagnosis of BIs would enable the attending physician to start antibiotic therapy only in cases of proven infection, and to not start antibiotherapy if infection is unlikely. In this context, several biomarkers have been validated, such as PCT, probably the most widely used [34]. It was first proposed as an early marker of infection, but it was then determined that PCT was more useful to determine when to stop antibiotic therapy (antibiotic exposure can be reduced by three to seven days [6]) than for diagnosing infections. Nevertheless, the results of biomarker-guided therapies are not immediately available (results are available after hours or days) and the average cost for a single PCT analysis is high [6]. Several other biomarkers have been investigated in the field of early diagnosis of sepsis. Sensitivities and specificities of over 90% have been reported for 5 of them: interleukin IL-12 [35], interferon-induced protein 10 [36], group II phospholipase A2 [37], CD64 [38] and neutrophil CD11b [39]. However, aside from pediatric or bacteriemic patients, IL-12 [35], interferon-induced protein 10 [36], CD11b [39] and group II phospholipase A2 [37] have never been evaluated for diagnosis of VAP in adult patients. Interestingly, in a clinical trial involving 135 patients with confirmed viral or BIs, Nuutila *et al.* [38] reported a high sensitivity (94%) and specificity (98%) for CD64 in distinguishing febrile infections from healthy volunteers. Finally, even if some cytokines, such as TNF and IL-10 [40], are produced rapidly after the onset of sepsis, most of these proteic biomarkers need few hours to be generated, secreted and then, detected. By contrast, pathogen-induced metabolic changes in PMNs, such as respiratory burst, are fast, and the resulting changes in autofluorescence intensity are detectable within an hour. PCR technology is another biomarker approach which is available at bedside and detects pathogen DNA. It is an easy method with commercial kits, but like PCT, it is expensive and diagnosis is not immediately available. Also, contamination of samples can give “false- positive results” in 0.6 to 6% of the cases [41].

Our strategy based on autofluorescence variations in PMNs can help to diagnose or excluding BIs, in conjunction with other clinical and bacteriological methods. The method is especially attractive since it provides results in one hour. Furthermore, we standardized the cell fixation technique in a very simple, fast, and reproducible way and confirmed that is possible to use a classical epifluorescence microscope which can easily be applied in a regular hospital bacteriology laboratory.

One of the limitations of the study is that our new optical diagnosis tool, like most biomarkers for sepsis, could provide false-positive and false-negative results. Our clinical data should be interpreted with caution. The small number of cases does not make it possible to report accurate sensitivity or specificity. Further large scale clinical studies are warranted before drawing a definitive conclusion. Second, the criteria used for the diagnosis of VAP could impact the results of the present study. Clinical pulmonary infection score is not superior to conventional clinical criteria [42]. However it is useful for the decision to initiate probabilistic antibiotherapy [43] and for using short course antibiotic therapy in patients with a low likelihood of pneumonia [44]. Another limitation is that the present study is limited to a mouse model and human pneumonia, i.e. an autofluorescence signal provided mainly from PMNs. The next step for developing this new tool will be to investigate other *in vivo* infected clinical samples, such as those that are virally infected.

In summary, this study demonstrates that the autofluorescence of PMNs is helpful and easy to use, even in a routine microbiological laboratory setting. The immediate analysis of mean autofluorescence intensity of PMNs cytospined samples can detect a host-pathogen interaction as a surrogate marker of infection. In the future, this new technique could be used to detect changes in leukocyte metabolism, which could lead to the optimization of rapid infection diagnosis without the need to directly determine the pathogen or wait for bacterial culture results. Moreover, analysis of PMN autofluorescence could enhance infection diagnosis in the cases of failure of typical bacteriological laboratory techniques.

## Supporting Information

Figure S1 Evaluation of the neutrophil population in control and *Pseudomonas aeruginosa-*or *Staphylococcus aureus*-induced pneumonia mice. Proportions of neutrophils (Ly6G^pos^ CD11b^pos^) were assessed in murine BAL 24 hours after intra-tracheal instillation of saline solution (C), *Pseudomonas aeruginosa* (PA) or *Staphylococcus aureus* (SA). Boxes represent median (interquartile ranges) from 2 independent experiments (n≥5 mice per group). A Kruskal-Wallis test with Mann-Whitney test using Bonferroni's corrections for post-hoc analysis was used. *: p<0.001 compared with sham group.
(TIF)
