# Supplementary figures and images for: Analysis of Autofluorescence in Polymorphonuclear Neutrophils: A New Tool for Early Infection Diagnosis
Source: PLoS One. 2014 Mar 21;9(3):e92564. doi: 10.1371/journal.pone.0092564 (PMC3962417; doi:10.1371/journal.pone.0092564)

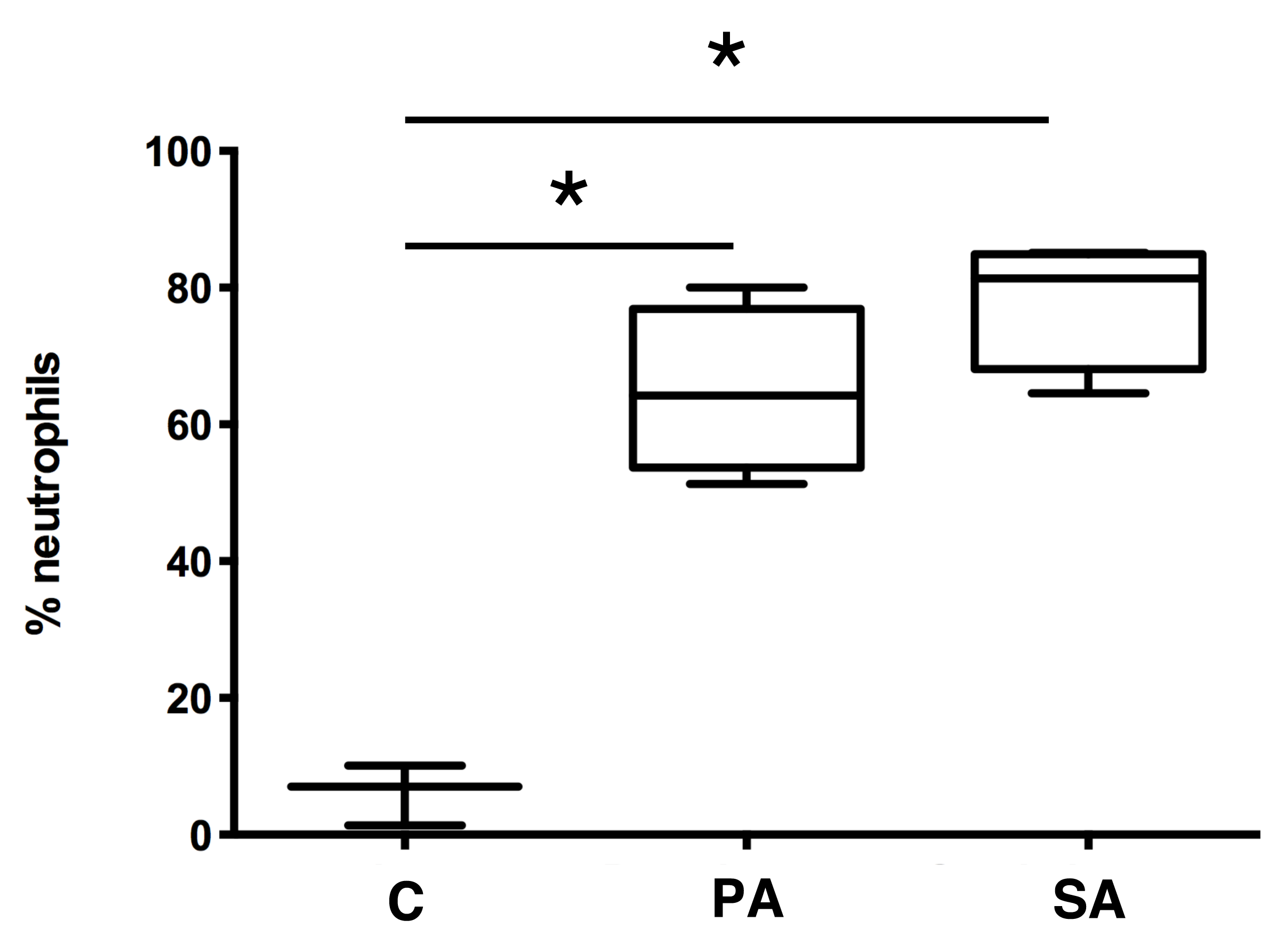

Supplement: Figure S1 — Evaluation of the neutrophil population in control and Pseudomonas aeruginosa-or Staphylococcus aureus-induced pneumonia mice. Proportions of neutrophils (Ly6Gpos CD11bpos) were assessed in murine BAL 24 hours after intra-tracheal instillation of saline solution (C), Pseudomonas aeruginosa (PA) or Staphylococcus aureus (SA). Boxes represent median (interquartile ranges) from 2 independent experiments (n≥5 mice per group). A Kruskal-Wallis test with Mann-Whitney test using Bonferroni's corrections for post-hoc analysis was used. *: p<0.001 compared with sham group. (TIF) [file pone.0092564.s001.tif]
